# Regulating the surface of anion-doped TiO_2_ nanorods by hydrogen annealing for superior photoelectrochemical water oxidation

**DOI:** 10.1186/s40580-022-00323-9

**Published:** 2022-07-19

**Authors:** Jongseong Park, Seonyong Lee, Tae Hyung Lee, Changyeon Kim, Sang Eon Jun, Ji Hyun Baek, Jae Young Kim, Mi Gyoung Lee‬, Sang Hyun Ahn, Ho Won Jang

**Affiliations:** 1 Department of Materials Science and Engineering, Research Institute of Advanced Materials, Seoul National University, Gwanak-ro 1, Seoul, 08826 Republic of Korea; 2 Department of Electrical and Computer Engineering, University of Toronto, 35 St. George Street, Toronto, ON M5S 1A4 Canada; 3 School of Chemical Engineering and Materials Science, Chung-Ang University, 84 Heukseok-ro, Dongjak-gu, Seoul, 06974 Korea; 4 Advanced Institute of Convergence Technology, Seoul National University, Suwon, 16229 Republic of Korea

**Keywords:** Photoelectrochemical, Water splitting, Nanostructures, Titanium dioxide, Hydrogen annealing

## Abstract

**Abstract:**

Dedications to achieve the highly efficient metal oxide semiconductor for the photoelectrochemical water splitting system have been persisted to utilize the TiO_2_ as the promising photoanode material. Herein, we report notable progress for nanostructured TiO_2_ photoanodes using facile sequential one-pot hydrothermal synthesis and annealing in hydrogen. A photocurrent density of 3.04 mA·cm^−2^ at 1.23 V vs. reversible hydrogen electrode was achieved in TiO_2_ nanorod arrays annealed in hydrogen ambient, which is approximately 4.25 times higher than that of pristine TiO_2_ annealed in ambient air. 79.2% of incident photon-to-current efficiency at 380 nm wavelength demonstrates the prominence of the material at the near-UV spectral range region and 100 h chronoamperometric test exhibits the stability of the photoanode. Detailed studies regarding crystallinity, bandgap, and elemental analysis provide the importance of the optimized annealing condition for the TiO_2_-based photoanodes. Water contact angle measurement displays the effect of hydrogen annealing on the hydrophilicity of the material. This study clearly demonstrates the marked improvement using the optimized hydrogen annealing, providing the promising methodologies for eco-friendly mass production of water splitting photoelectrodes.

**Graphical Abstract:**

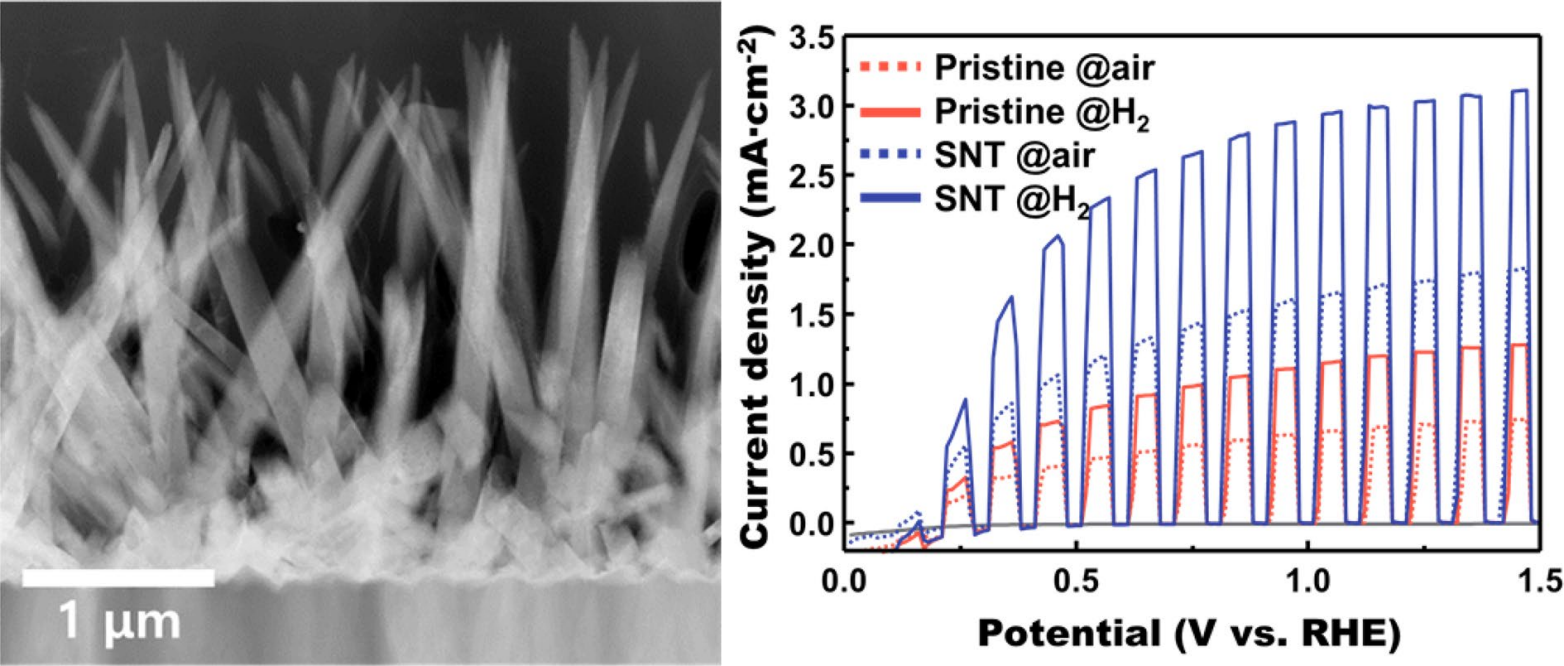

**Supplementary Information:**

The online version contains supplementary material available at 10.1186/s40580-022-00323-9.

## Introduction

Regulations aimed at reducing air pollution encourage a swift change to sustainable and alternative energy [1, 2]. Despite massive efforts to replace fossil fuels, they still account for 87% of total global energy consumption due to rapidly expanding demand [3]. As the annual increase in global mean surface temperature accelerates, intense research on the promising alternative energies becomes urgent further [4]. Hydrogen fuel, which has a 3 times higher energy density than gasoline, has long been seen as a possible future energy source. However, because the generation process uses fossil fuels and emits carbon dioxide and carbon monoxide, hydrogen fuel is hard to be regarded as a perfectly clean energy source. The discovery of the photocatalytic effect of TiO_2_ was a breakthrough in the manufacture of clean hydrogen fuel [5, 6]. The methodology of producing clean hydrogen fuel using sunlight and water has promoted tremendous studies. However, the high cost of renewable hydrogen production than fossil fuel-based production still hinders the commercialize of eco-friendly hydrogen fuel production [7].

Photoelectrochemical (PEC) water splitting has been the subject of extensive research in recent years [8–12]. The PEC water splitting cell, which outperforms the simple photocatalyst in solar-to-hydrogen conversion, has been focused as a promising clean hydrogen fuel production technology. Photoanode and photocathode compose the PEC water splitting cell, which corresponds to the n-type and p-type semiconductor materials, respectively. In the pursuit of developing photoelectrodes for the PEC water splitting cell, the photoanode with a complex reaction step involving four electrons achieved inferior results than the photocathode, a simple two-electron involving reaction [13]. Thus, to improve the overall efficiency of PEC cells, further dedications must be put into developing efficient photoanodes.

Since its discovery, TiO_2_ has been representative n-type semiconductor material with photocatalytic effect [5]. The significance of TiO_2_ as a commercially prominent material is due to its thermodynamic and chemical stability. To make the TiO_2_ a promising photoanode material, a large amount of research has been stated. Nanostructuring is the representative strategy to promote further improvement by enhancing photon absorption with enlarged specific surface area and internal reflection. Furthermore, in the case of one-dimensional structure such as nanorods [14–16] or nanotubes [17, 18], constraining the carriers’ pathway induce the fast charge migration. The early studies on one-dimensional (1D) nanostructured TiO_2_ successfully demonstrated a remarkable advance over the TiO_2_ thin film. Furthermore, it was claimed that the three-dimensional (3D) hierarchical nanostructure had an extraordinarily increased photon absorption property [19]. Doping was the other methodology to deal with the weakness of TiO_2_, which is large bandgap and low carrier mobility. Various studies about the anion dopants of B [20, 21], F [20], S [22, 23], and N [24] and cation dopants of C [25], H [26], Fe [27, 28], Cr [27], Sn [29], and Ta [30] successfully exhibit the further progress in PEC performance by improving the carrier concentration, carrier mobility, and the bandgap narrowing. The approaches described above showed drastic advances compared to the pure TiO_2_ thin film. Nevertheless, when compared to the other promising photoanode materials such as WO_3_ [31, 32] and BiVO_4_ [33], TiO_2_ still needs further progress to be utilized.

Recently, the studies controlling the intrinsic defect via surface engineering or the non-stoichiometric synthesis are intensively researched in the TiO_2_ photocatalysts field. Early studies demonstrated that the defect levels inside the bandgap effectively modify the electronic state of the TiO_2_, being able to absorb photons from a wider range of wavelength. The defect engineered anatase TiO_2_ photocatalysts are referred to as the black [34] and blue [35, 36] anatase depending on their colors. The anion-deficient TiO_2_ successfully manifested a strong visible light absorption than the pure TiO_2_ [34–36]. Black and blue anatase were synthesized under various conditions such as annealing under H_2_ atmosphere, solvothermal process with an organic solvent, and using a reducing agent. However, exactly controlling the chemical state and spatial distribution of electronic state modifier still remains as the unexploited room to explore.

In this work, notably efficient TiO_2_ based photoanodes have been synthesized by an all-solution process. Photoanodes manifesting 4.25 times higher photocurrent density than the pure TiO_2_ nanorod arrays have been achieved by incorporating all the strategies above. The combination of the facile one-pot hydrothermal synthesis including the sulfur and nitrogen precursor and the annealing under the H_2_ atmosphere accomplished the remarkable advance of photocurrent density. Chronoamperometric measurement for 100 h exhibits the excellent reliability of these photoanodes. Also, the importance of the all-solution process has to be emphasized as the initial facility cost of the PEC water splitting system could not be neglect due to the large area of photoelectrodes [37–40]. Thus, a scalable all-solution process gets expectations as the future production method for mass production. The crystallographic, elemental, and bandgap analysis depending on the H_2_ annealing during the optimizing H_2_ annealing condition reveals how surface disorder optimization importantly affects PEC performance of photoelectrodes. Even the photocurrent density, electrochemical impedance, and incident photon-to-current density will be stated, showing the distinctive improvement of this material. Finally, the contact angle exhibits the effect of oxygen vacancies on the adsorptive power to the water molecule and hydroxyl groups.

## Methods/experimental

### Preparation of various TiO_2_ nanorod arrays and TiO_2_ thin film

All reagents in this process were employed without further purification. Both pristine TiO_2_ nanorod arrays and S, N-doped TiO_2_ nanorod arrays were synthesized by a facile hydrothermal process. Polycrystalline fluorine-doped tin oxide (FTO) thin film deposited glass substrates were prepared by sequential cleaning with the polar and non-polar solvent. In the order of acetone, isopropanol, and distilled water (18.3 MΩ·cm), the FTO substrates were cleaned for 30 min using ultrasonic cleaner (Branson CPX, Emerson). Simultaneously, the aqueous solutions for the hydrothermal process were prepared. 2.71 mmol of Titanium chloride (> 99.9%, TiCl_4_, Sigma-Aldrich), and 28 ml of hydrochloric acid (35%, HCl, Daejung Chemicals) were added to the 22 ml of distilled water and vigorously stirred until the solution becomes completely obvious. For the S, N-doped TiO_2_ nanorod arrays, 0.257 mmol of sulfamic acid (> 99%, H_3_NSO_3_, Sigma-Aldrich) was additionally employed in the hydrothermal precursor solution. The prepared FTO substrates and solutions were transferred to the 100 ml of Teflon vessel, while the FTO substrates’ active surface faces up. The precursor solution containing Teflon vessel was lined into the stainless steel autoclave and sealed tightly, and was heated at 180 ℃ for 6 h using the electronic oven. After the natural cooling down to room temperature in the oven, the as-grown pristine TiO_2_, and S, N-doped TiO_2_ nanorod arrays were repeatedly washed and dried with distilled water and N_2_ gas to remove the residual impurities. The rinsed samples were annealed under ambient air at 350–550 ℃ for 1 h. Following heat treatment under H_2_ atmosphere was carried out for the H_2_-annealed samples. During the heat treatment, all samples were kept inside the furnace while the temperature falls to room temperature. Finally, various pristine TiO_2_ and S, N-doped TiO_2_ nanorod arrays samples were obtained when removed from the furnace.

The TiO_2_ thin films for contact angle measurement also prepared by electron beam evaporator (e-beam Korea Vacuum Tech Co., Ltd.). Same with the preparation for the hydrothermal process, sequential substrates cleaning process prepares the FTO substrates. Approximately 100 nm thickness of TiO_2_ thin films were deposited on the FTO substrates, while the thickness monitor in the electron beam evaporator system examines the samples’ thickness. The as-deposited samples were heated at 350 ℃ for 1 h under the ambient air and H_2_ atmosphere, respectively. The samples were finally obtained after the natural cooling to room temperature in the furnace.

### Materials characterization

The glazing incident X-ray diffractometer (GIXRD; X’pert pro, PANalytical) clarifies the crystal structure of the samples. Field emission scattering electron microscopy (FE-SEM; MERLIN Compact, ZEISS) examines the nanostructure of the nanorod arrays. Furthermore, detailed nanostructure, crystallographic analysis, and elemental mapping were carried out using transmission electron microscopy (TEM; JEM-2100F, JEOL), and energy dispersive spectroscopy (EDS). The further elemental analysis was operated by an X-ray photoelectron spectrometer (XPS; AXI Supra, KRATOS). The obtained full spectra of samples were analyzed and deconvoluted using Casa XPS software. UV-vis spectroscopy (V-770, JASCO) measures the optical reflectance of the samples, which was used for the bandgap calculation. A contact angle meter (DSA 25, Kruss) investigates the contact angle between the TiO_2_ thin film and electrolyte. Distilled water and 1 M NaOH was tested as the contact angle electrolyte.

### Measurement of photoelectrochemical performance

The three-electrode system operated by computer-controlled potentiostat (Nstat, Ivium Technologies) performs the oxygen evolution reaction measurements. Saturated Ag/AgCl reference electrode, platinum mesh counter electrode, and working electrode composes the three-electrode system. All electrodes were submerged in the 1 M NaOH standard solution (pH = 13.6) containing quartz vessel during the PEC performance measurements. While the Xenon Arc lamp (mW·cm^−2^) irradiates the light to the working electrode, the linear sweep voltammetry (LSV) was performed under a potential from 0 to 1.5 V vs. RHE. To determine the potential versus RHE, following Equation (1) was used:

1 $${\text{E}}_{{{\text{RHE}}}} = {\text{E}} + {\text{E}}_{{{\text{Ag}}/{\text{AgC}}l}} + 0.059 \times {\text{pH}}$$

where E_Ag/AgCl_ is 0.198 V and E is applied potential vs. Ag/AgCl electrode [41]. Electrochemical impedance spectroscopy (EIS) were measured under same irradiation condition. Under the constant 1.23 V vs. RHE of potential, Nyquist plot was obtained by EIS measurement in the frequency range of 10 Hz–250 kHz. Using the light source and the monochromator (MonoRa150), incident photon-to-current efficiency (IPCE) measurement determines the external quantum efficiency of the photoanodes under the 1.23 V vs. RHE potential condition. Mott-Schottky (M-S) plot was used to determine the carrier concentration of each photoanode. To obtain carrier concentration, calculation using M-S plot was conducted using following Equation (2):

2 $$\frac{1}{{C_{{{\text{sc}}}}^{2} }} = \frac{1}{{\varepsilon_{{\text{r}}} \varepsilon_{0} {\text{A}}^{2} {\text{e}}N_{{{\text{Dopant}}}} }}\left( {E - E_{{{\text{fb}}}} - \frac{{{\text{kT}}}}{{\text{e}}}} \right)$$

where C_sc_ is the capacitance of the space charge layer of the semiconductor electrode, ε_r_ is relative permittivity, ε_0_ is vacuum permittivity, A is the surface area of the electrode, e is an elementary charge, N_dopant_ is the carrier concentration, E is the applied potential, E_fb_ is flat-band potential, k is a Boltzmann constant, and T is the temperature. M-S curve was obtained by M-S measurement performed in the potential range of 0–1.0 V vs. RHE [41]. In order to analyze the surface area of the photoanodes, the optical images of TiO_2_ photoanodes are investigated using the Adobe Photoshop.

## Results and discussion

**Fig. 1:**
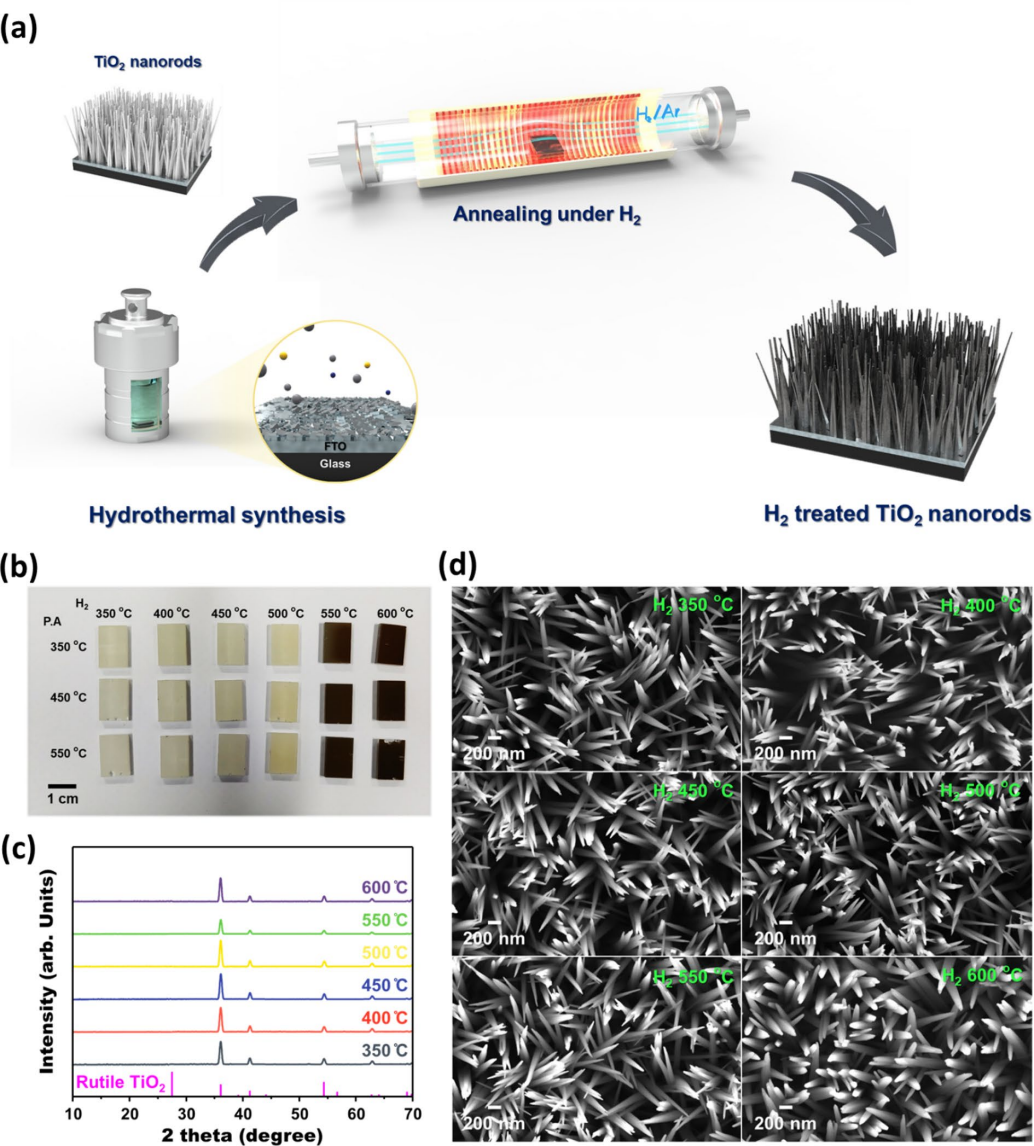
**a** Schematics illustrating the facile procedure of H_2_ treated S, N-doped TiO_2_ nanorod arrays. **b** Optical image of the S, N-doped TiO_2_ nanorod arrays under the various annealing condition. **c** XRD spectra and **d** SEM images for the 350 °C post-annealed S, N-doped TiO_2_ samples under various H_2_ treatment temperature. The XRD spectra were indexed using the JCPDS crystallographic database. (#00-021-1276)

**Fig. 2:**
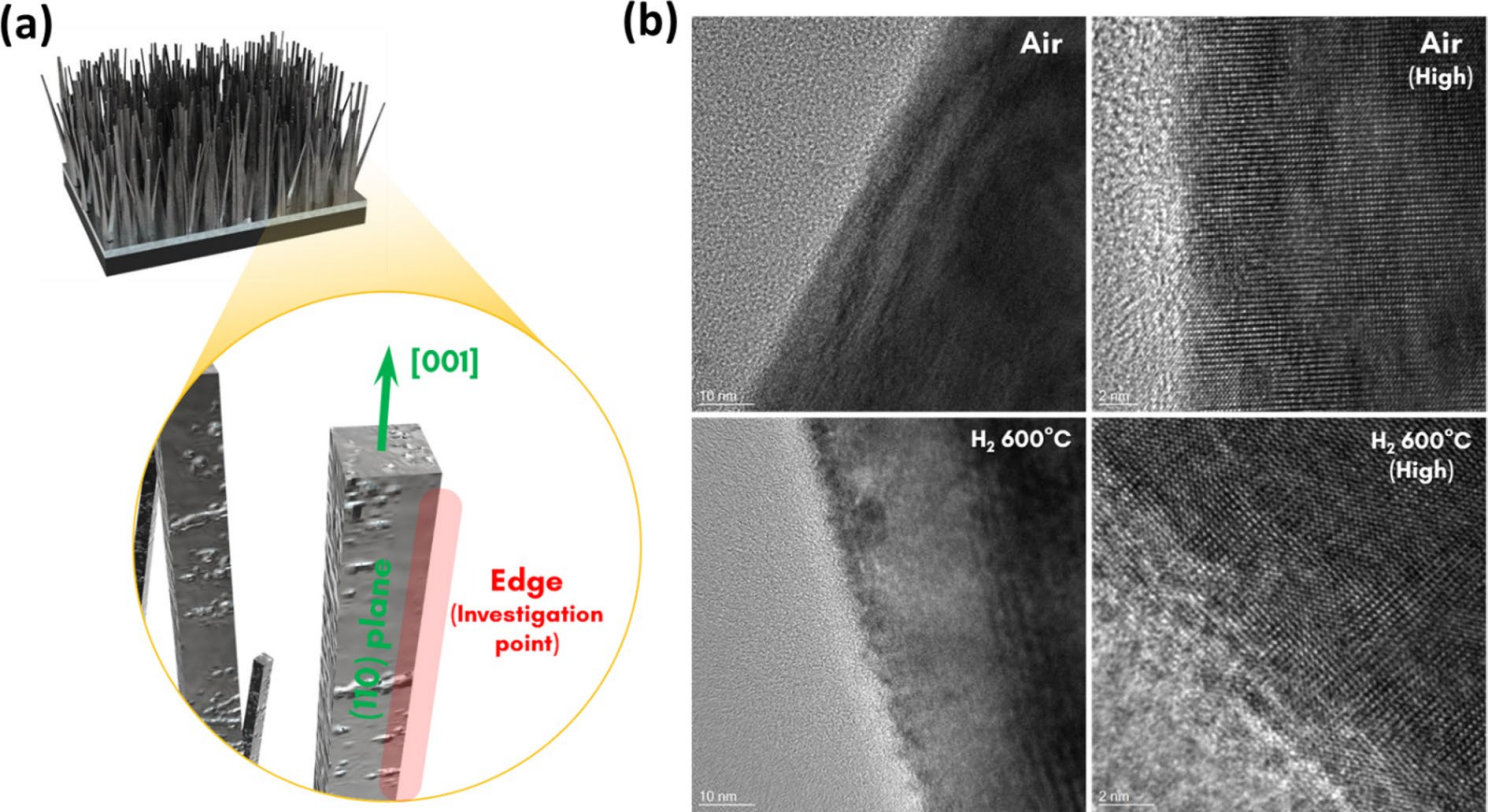
**a** Schematics depicting the investigation point of the TiO_2_ nanorods. The edge of the square-shaped nanorod, having the (110) plane as the side plane, was determined to clarify the exact crystallographic state of the surface. **b** TEM and HRTEM images of S, N-doped TiO_2_ nanorods annealed under air at 350 °C and H_2_ at 600 °C, respectively

As illustrated in Fig. 1a, the H_2_-treated S, N-doped TiO_2_ nanorod arrays were synthesized by a considerably facile process. Sequential annealing in air and hydrogen ambient was controlled to test the optimum annealing condition. An optical image of the S, N-doped TiO_2_ nanorod arrays under various conditions is shown in Fig. 1b. Below 500 ℃ of H_2_ annealing temperature, no significant color change occurs. The colors of the specimen drastically darken as the H_2_ annealing temperature rises beyond 500 ℃, which was a similar temperature with the previous studies dealing with the black TiO_2_ nanoparticles [34, 42, 43]. Thus, H_2_ annealing effectively applied to the rutile phase TiO_2_ not even for the anatase phase of previous reports. The X-ray diffraction (XRD) spectra of the samples annealed under 350 ℃ of post-annealing temperature were investigated to check the crystallinity. As shown in Fig. 1c, the XRD patterns of each sample were almost same regardless of H_2_ annealing condition and color, which means there was no significant crystallinity change during annealing at the overall matrix. The microstructure was also unchanged after the annealing when investigated by scanning electron microscope (SEM), except for the 600 ℃ of H_2_ annealing temperature. SEM images in Fig. 1d shows that the nanorods under the 600 ℃ of H_2_ annealing condition are blunter than other samples. Thus, further investigation was carried out using TEM to exactly examine the surface having black color. To prevent the confusion due to the crystallinity of inner matrix, the edge of the nanorods was examined to verify the surface condition exactly, as illustrated in Fig. 2a. The HRTEM analysis figures out the surface condition of the S, N-doped TiO_2_ nanorods annealed under the air and H_2_. As displayed in Fig. 2b, the H_2_ annealed sample has a roughened surface compared to the air-annealed sample. Also, crystallinity at the roughen surface shows a disordered state which is 1–2 nm thickness, as demonstrated in HRTEM image. Therefore, analogous to the previous studies concerning black TiO_2_ photocatalysts, the origin of the black color comes from the disordered and roughened surface [34, 43].

**Fig. 3:**
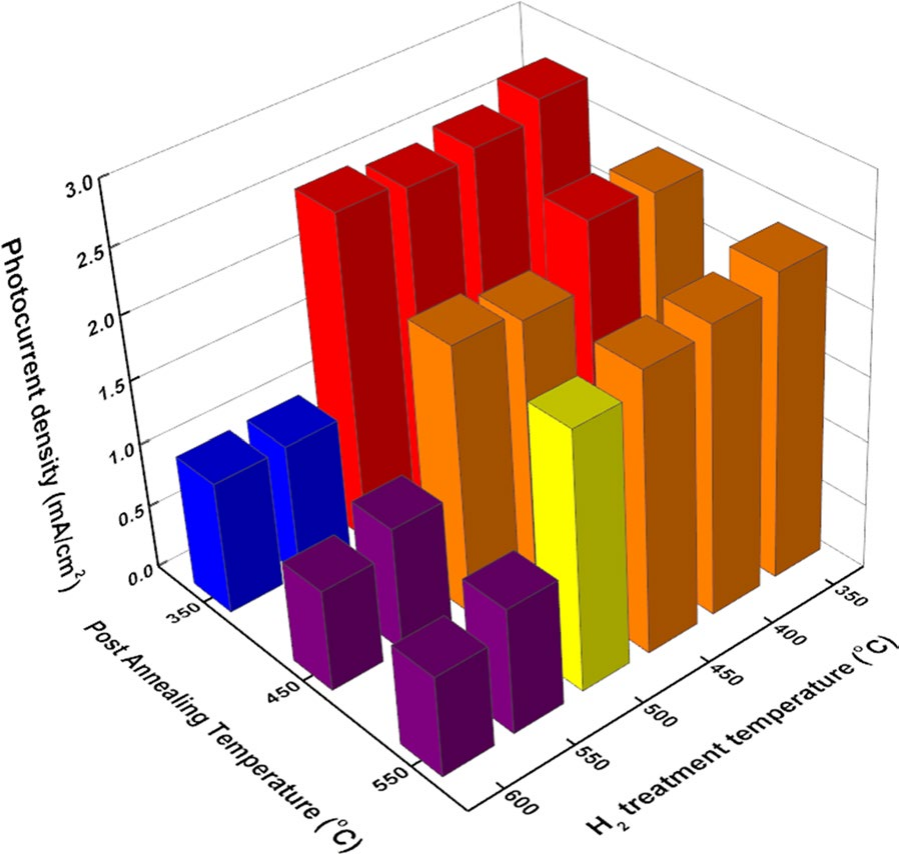
The photocurrent density comparison graph for the post annealing and H_2_ treatment temperature. The photocurrent densities were determined at the 1.23 V vs. RHE condition. The color of bar designates follows: > 2.5: red, > 2.0: orange, 1.5 > yellow, 1.0 > blue, 0.5 > purple

To explore the optimum condition for the PEC photoelectrodes, photocurrent density at the 1.23 V vs. RHE was compared in Fig. 3. Total LSV curves of all samples, including the air-annealed sample, were depicted in Additional file 1: Fig. S1. Although most of samples show notable increment than the pristine TiO_2_ nanorods sample, the severe decrease of photocurrent density was shown over 550 ℃ of H_2_ annealing, which corresponds to the black colored samples having rough and disordered surface. 350 ℃ post-annealed samples exhibit slightly better performance than the others. Considering the previous work dealing with black TiO_2_ photocatalysts, black TiO_2_ usually shows better catalytic performance than the pristine samples. However, in case of photoelectrodes study, it can be concluded that too much amount of surface disorder hinders the PEC performance.

**Fig. 4:**
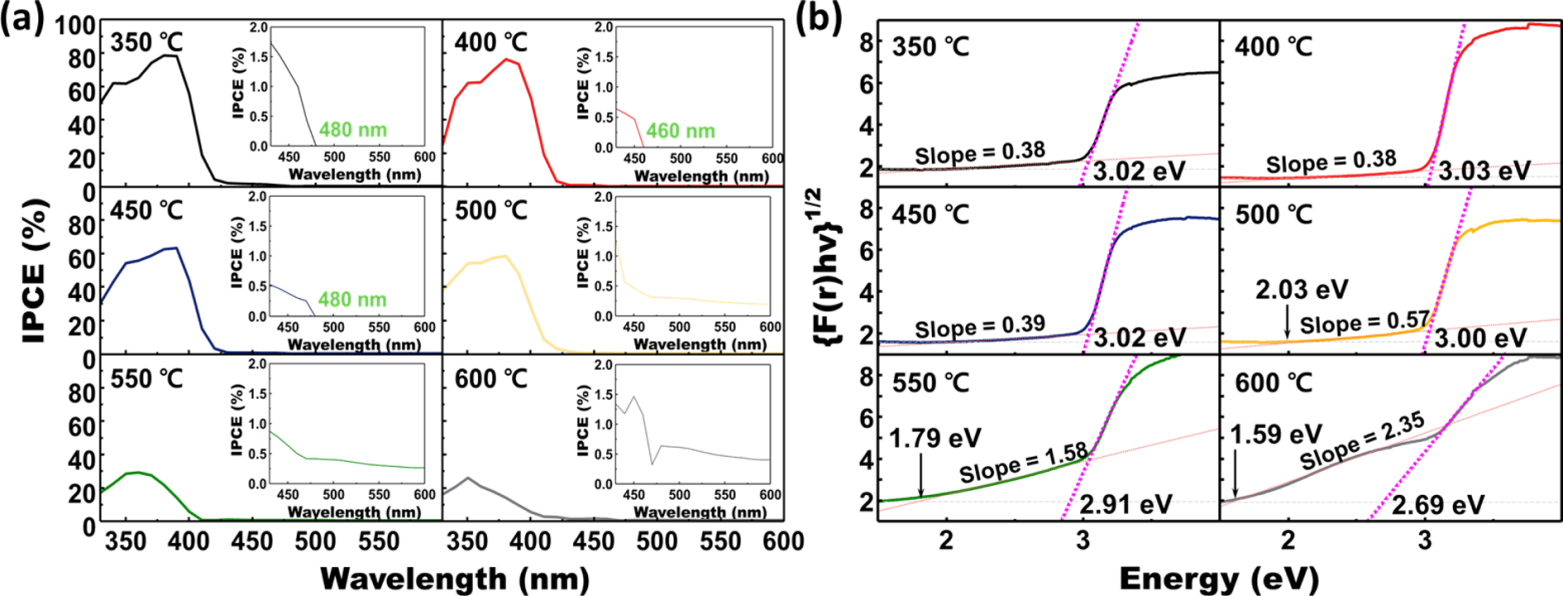
Bandgap analysis for the 350 °C air-annealed S, N-doped TiO_2_ under various H_2_ treatment conditions. **a** The results of IPCE measurement for S, N-doped TiO_2_ photoanode. The current was obtained under 1.23 V vs. RHE condition. **b** Kubelka–Munk plots for S, N doped TiO_2_ nanorod arrays under various H_2_ treatment conditions. The plots were calculated by the reflection for the wavelength, obtained from UV–vis measurement

Further investigations such as the bandgap analysis and elemental analysis were performed to research the materials’ change during the H_2_ annealing. IPCE and UV-vis analyze the influence of H_2_ annealing temperature on the bandgap. The results of the IPCE measurement are demonstrated in Fig. 4a. The bandgap annealed below 450 ℃ was not significantly changed. The maximum photon absorption wavelength was around 480 nm, which corresponds to the 2.58 eV. This value also corresponds to a maximum theoretical photocurrent density of 5 mA/cm^2^ [44], which shows quite a smaller bandgap compared to 3.0 eV the band gap of the pure rutile TiO_2_ due to the doping and H_2_ annealing [23, 24, 34, 42]. The absorbance range changes drastically over 500 ℃ of H_2_ annealing temperature. The IPCE did not become zero above 480 nm of wavelength, showing the photon absorption at a longer wavelength. Therefore, it can be suspected that the high temperature H_2_ annealing distinctively drives the narrow bandgap, which can be observed by the black color of the sample [45, 46]. However, very low value of IPCE investigated at a long wavelength meaning the poor external quantum efficiency. Similar results were found by UV-vis analysis, as displayed in Fig. 4b. UV-vis results also show significant change over 500 ℃ of H_2_ annealing temperature. As the annealing temperature rises, the slope becomes steep, which is marked in the graph. In other words, H_2_ annealed S, N-doped TiO_2_ nanorods also absorb light in an energy region smaller than the bandgap of pristine rutile TiO_2_. Similar to the previous studies and IPCE results in this work, it means that the surface disorder drives the narrow bandgap by generating the defect level inside the bandgap [24, 34]. And, the narrow bandgap by the defect level effectively contribute to the photon absorption. However, considering the photocurrent density results shown in Fig. 3, the optimum H_2_ annealing condition seems needed even though the advance of photon absorption.

**Fig. 5:**
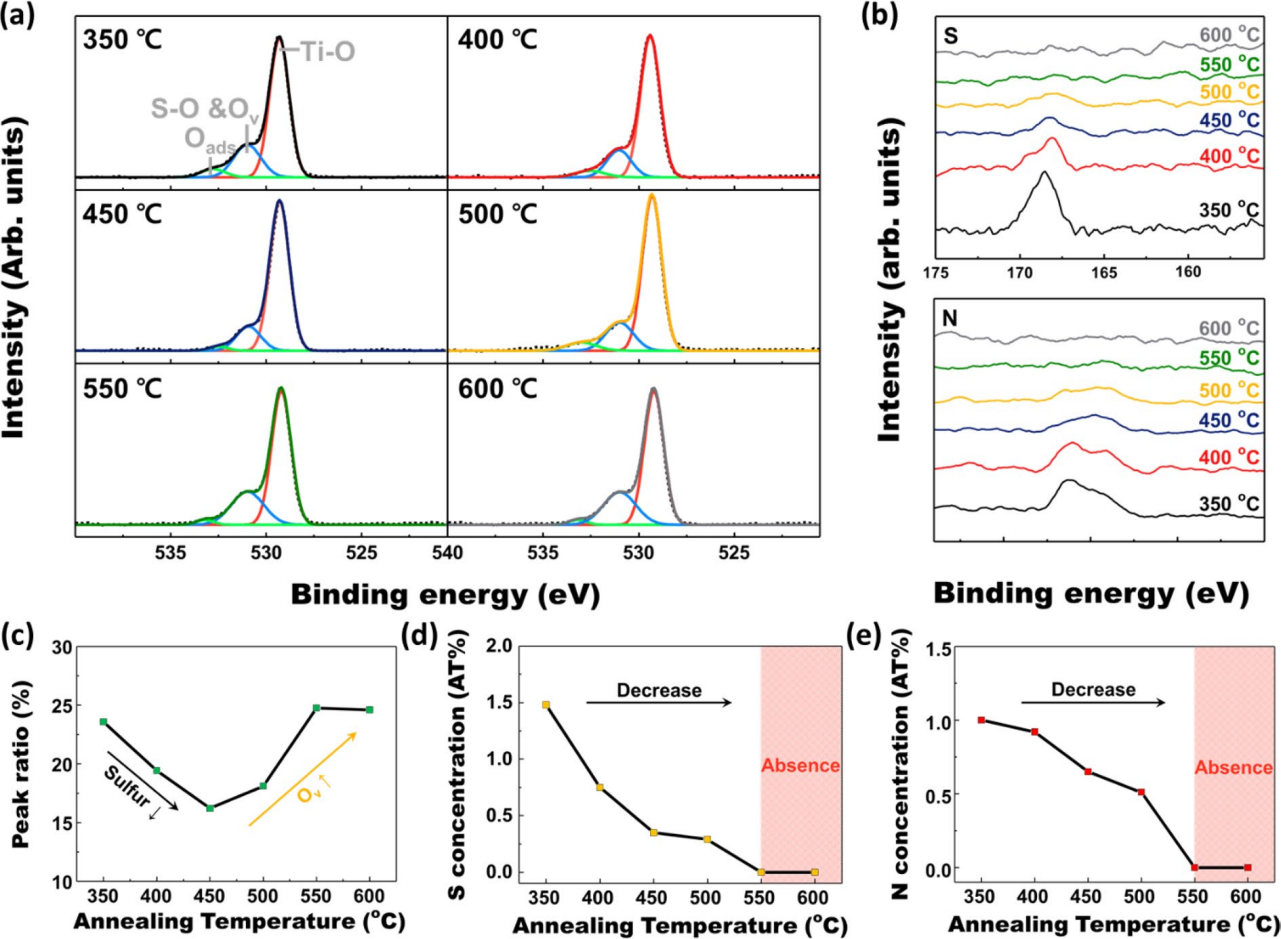
Elemental analysis by XPS spectra. **a** O 1 s, bb S 2p and N 1 s peaks for the S, N-doped TiO_2_ nanorod arrays under various H_2_ treatment conditions. **c** Change of peak volume ratio (S–O and O_v_/Ti–O) for H_2_ annealing temperature. **d** Sulfur and **e** nitrogen dopant concentration change for H_2_ annealing temperature, obtained by XPS analysis

Notable elemental change at the O 1s, S 2p, and N 1s peaks were also investigated by XPS measurement, shown in Fig. 5. O 1s peaks were deconvoluted to clarify the existence of oxygen vacancies, shown in Fig. 5a. O 1s peaks can be divided into Ti–O bond peak, S–O bond and oxygen vacancy (S–O and O_v_) peak, and surface adsorption peak, respectively. As the oxygen vacancy peak overlaps with the S–O bond peak, sulfur concentration also has to be considered to identify the amount of oxygen vacancy [47]. Therefore, S 2p and N 1s peaks are also demonstrated in Fig. 5b. It can be noticed that the sulfur and nitrogen concentration in the TiO_2_ matrix severely drops until the 550 ℃ of H_2_ annealing temperature. The sulfur and nitrogen dopants in the TiO_2_ matrix disappear above the 550 ℃ of H_2_ annealing temperature. The rest of the XPS spectra depicting the Ti 2p peaks are demonstrated in Additional file 1: Fig. S2. To easily discuss the oxygen vacancy, the peak volume ratio between S–O and O_v_ peak and the Ti–O bond peak is calculated and arranged in a single graph, Fig. 5c. The graphs about sulfur and nitrogen concentration are also demonstrated in Fig. 5d and e, respectively. The peak volume ratio shows a V-shape curve meaning that the change of S–O and O_v_ peak has the inflection point. Considering the sulfur concentration that decreases and diminishes at high annealing temperature, the inflection point shows that the number of oxygen vacancies was not significantly increased below 450 ℃ of H_2_ annealing temperature. The drastic rise of oxygen vacancies, demonstrated by the increasing peak volume ratio, occurs over 500 ℃ of H_2_ annealing temperature [48]. As a result, it can be noticed that the H_2_ annealing at high temperature develops dopant loss at the surface and creates too many oxygen vacancies. The dopant loss and oxygen vacancies lower the crystallinity of the surface and acts as a recombination site of charge carriers [49, 50], interfering with the overall PEC performance. Based on the results above, owing to suitable bandgap alignment and defect site concentration, the optimum H_2_ annealing temperature for S, N-doped TiO_2_ nanorod arrays were set as 350 ℃.

**Fig. 6:**
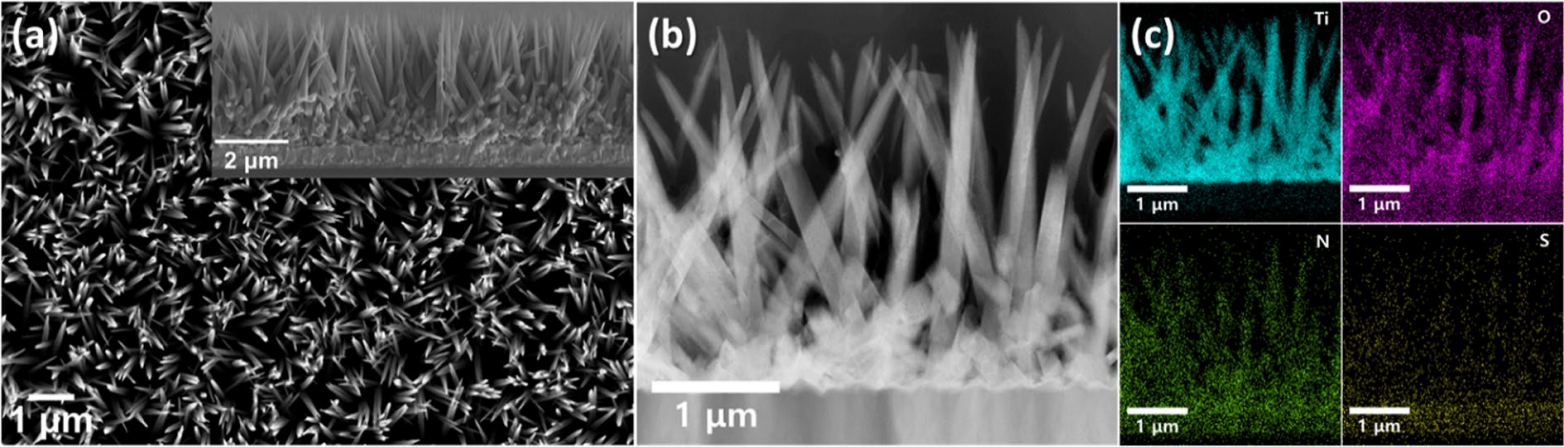
Nanostructure of S, N-doped TiO_2_ nanorod arrays annealed under H_2_ atmosphere at 350 °C, selected as the optimum H_2_ treatment condition. **a** Planar view obtained by FESEM. Cross-sectional view is also displayed by inset image. **b** Planar view investigated by STEM and **c** corresponding EDS elemental mapping results

As depicted in Fig. 6, SEM and TEM investigated the nanostructure of the optimum sample. The SEM images of the pristine TiO_2_ sample annealed under air and H_2_ at 350 ℃ and S, N-doped TiO_2_ sample annealed under air at 350 ℃, which are prepared for the reference samples, are demonstrated in Additional file 1: Fig. S3. Approximately 3–4 μm length nanorods have 50–100 nm of diameter, having a thinner size than the pristine TiO_2_. It makes a much larger specific area than the pristine TiO_2_, supporting the photon absorption of photoelectrodes [51–53]. Also, EDS performs the elemental analysis of optimum H_2_-treated S, N-doped TiO_2_ sample. The conformal distribution of sulfur and nitrogen dopant in the TiO_2_ matrix is investigated in Fig. 6c.

**Fig. 7:**
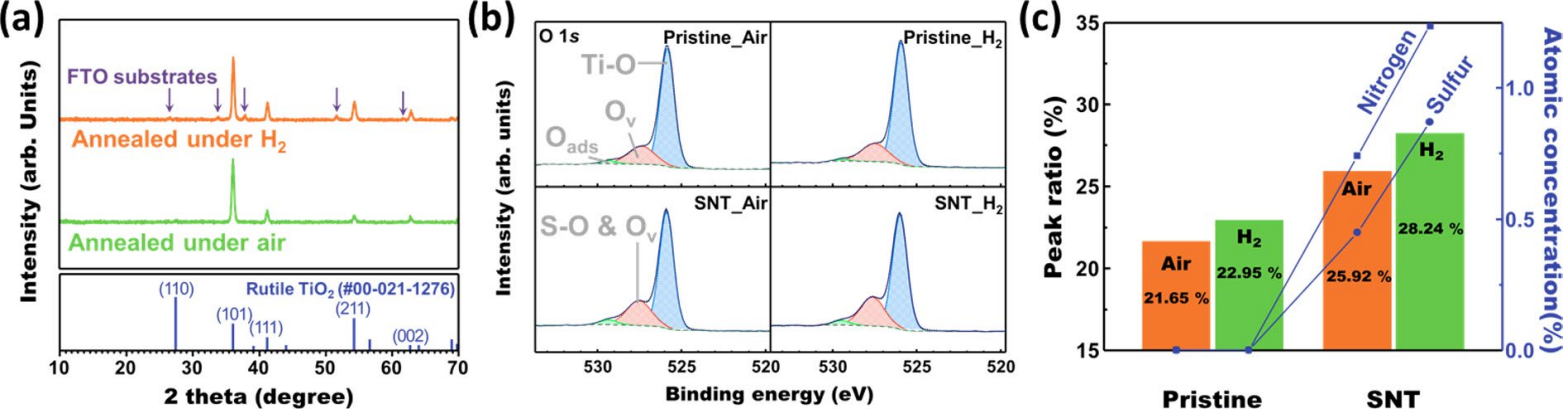
**a** XRD spectra of the S, N doped TiO_2_ nanorod arrays annealed under ambient air and H_2_ atmosphere at 350 °C. **b** O 1 s XPS spectra of the optimum and reference samples. **c** Comparison chart including the Peak volume ratio between the S–O and O_v_ and Ti–O bonding peak and the dopant concentration for the optimum and reference samples

XRD and XPS carried out the crystallographic and elemental analysis, respectively. XRD spectra in Fig. 7a shows the crystallographic consistency after the doping and H_2_ annealing. Without peak shift compared to the rutile TiO_2_ phase, only the preferential peaks appear due to the preferential growth long [001] direction [54, 55]. XPS spectra of each sample are shown in Fig. 7b. XPS spectra for the Ti 2p, S 2p, and N 1s are shown in Additional file 1: Figs. S4, S5. To perform the elemental analysis, the peak volume ratio between S–O and O_v_ peak and the Ti–O bond peak was considered to identify the effect of optimum H_2_ annealing condition on the oxygen vacancy concentration, in good agreement with Fig. 5. The calculated peak ratio, and sulfur and nitrogen concentration for the doping and annealing condition are merged in Fig. 7c. With H_2_ annealing, the peak volume ratio rises from 21.65 to 22.95% for pristine TiO_2_, and 25.92–28.24% for S, N-doped TiO_2_ sample. It can be noticed that the H_2_ annealing arises the oxygen vacancy concentration in both cases. Unexpectedly, dopant concentration also arises after the H_2_ annealing. When considering the XPS result as the surface analysis because of the 5 nm of scanning depth of the XPS analysis, the oxygen loss at the surface can drive the migration of dopant in the TiO_2_ matrix toward the surface.

**Fig. 8:**
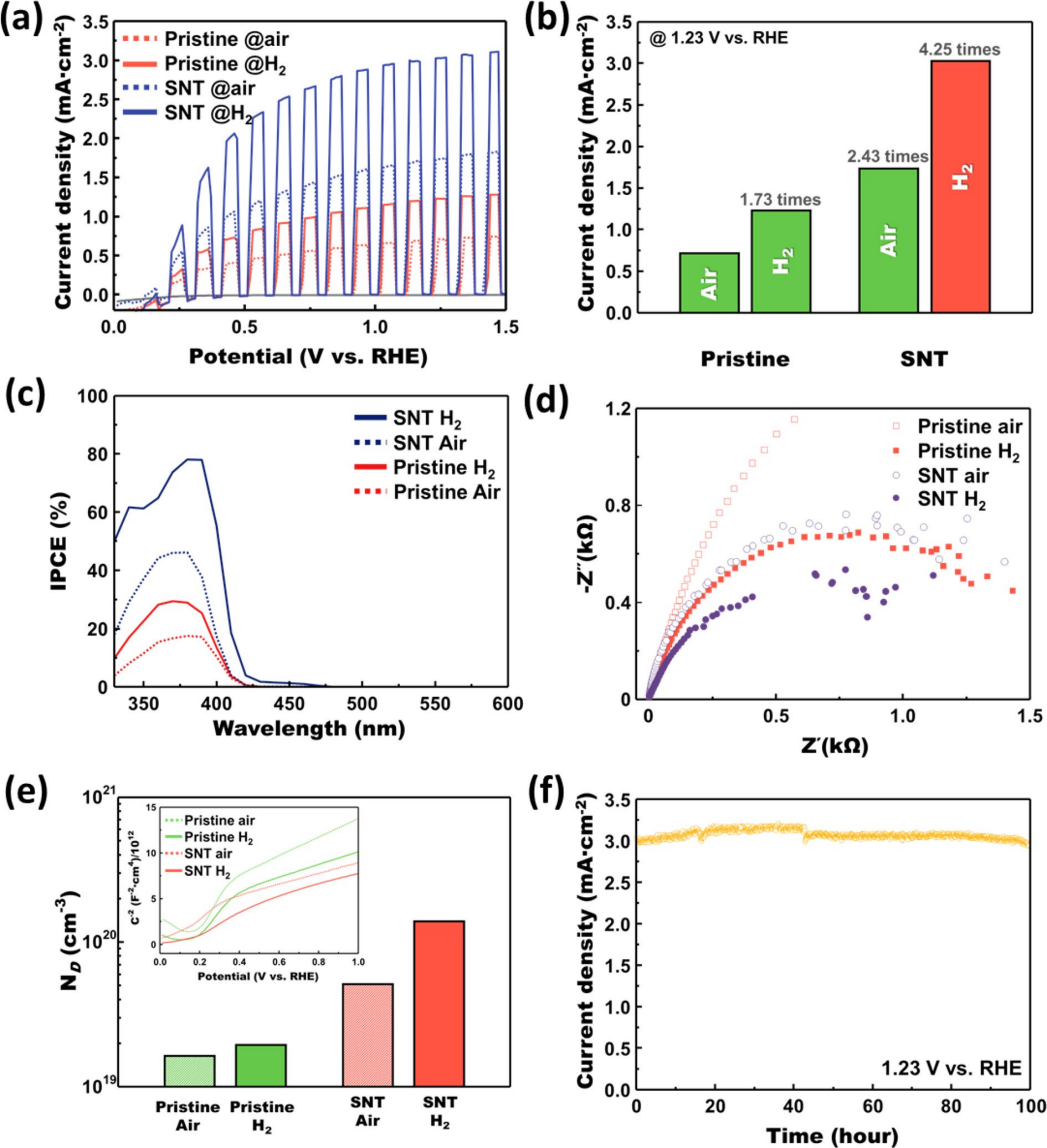
Determination of the photoelectrochemical properties. **a** Linear Sweep Voltammetry curves of the optimum and reference samples. **b** Comparison charge of the photocurrent density under the 1.23 V vs. RHE condition. **c** Results of incident photon-to-current efficiency measurement under 1.23 V vs. RHE condition. **d** Nyquist plots, obtained by electrochemical impedance spectroscopy, showing the charge transfer resistance of each sample. **e** Plot of the carrier concentration calculated by fitting the Mott-Schottky plots of each sample. Mott-Schottky plots of each sample also displayed as the inset image. **f** Chronoamperometric measurement at 1.23 V vs. RHE for 100 h

The PEC performance of the optimum H_2_ annealed sample and reference samples are determined and compared. Figure 8a shows the LSV curves of various samples under the chopping irradiation condition. Additionally, non-annealed samples’ LSV was also compared in Additional file 1: Fig. S6. Poor photocurrent density of the non-annealed sample exhibits the importance of heat treatment, and crystallinity for the PEC performance. The H_2_ annealed S, N-doped TiO_2_ sample exhibits outstanding PEC performance compared to the reference samples. It shows 3.04 mA/cm^−2^ of photocurrent density at 1.23 V vs. RHE, which is 4.25 times higher photocurrent density than the pristine TiO_2_ annealed under the air. Furthermore, in both doped and un-doped cases, H_2_ annealing boosts photocurrent density about 1.7 times higher than the annealing under the air. The photocurrent density of each sample at the 1.23 V vs. RHE is compared in Fig. 8b.

In Fig. 8c, IPCE measurements are displayed. IPCE measurement confirms the higher efficiency at the overall wavelength. By this measurement, the efficiencies of three fundamental processes associated with PEC can be easily considered:

3 $${\text{IPCE}} = \eta_{{{\text{e}} - /{\text{h}} + }} \times \eta_{{{\text{transport}}}} \times \eta_{{{\text{interface}}}}$$

where *η*_e-/h+_, *η*_transport_, and *η*_interface_ are defined as the electron-hole pairs generated per incident photon, the charge transport to the interface, and the charge transfer efficiency at the interface, respectively. Also, the IPCE can be expressed by a function of wavelength:

4 $${\text{IPCE}}\left( {\uplambda } \right) = \left| {{\text{j}}_{{{\text{ph}}}} \left( {{\text{mA}}\cdot{\text{cm}}^{ - 2} } \right)} \right|hc/P_{{{\text{mono}}}} \left( {{\text{mW}}\cdot{\text{cm}}^{ - 2} } \right){\uplambda }$$

where *j*_ph_ is the photocurrent density, *h* is the Planck’s constant, *c* is the light speed, and *P*_mono_ is the monochromated illumination power density [41]. H_2_-treated S, N-doped TiO_2_ shows 78.16 % efficiency at 380 nm of wavelength, which is the highest efficiency among all wavelengths. The highest efficiency of pristine under air, pristine under H_2_, and S, N-doped TiO_2_ under air are 17.64 %, 29.47 %, and 46.12 % respectively. Thus, the increments of IPCE well match with the results of LSV curves. Furthermore, 79.2 % of incident photon-to-current efficiency at 380 nm wavelength demonstrates the prominence of these materials at the near-UV spectral range region. EIS measurement, as shown in Fig. 8d confirms the effect of H_2_ annealing on the charge transfer resistance. The results of the charge transfer resistance calculation are stated in Table 1. The smaller half-circle in the Nyquist plot shows that the charge transfer resistance drops distinctively when applied doping and H_2_ annealing. Based on the previous studies about the relationship between the oxygen vacancy and water and hydroxyl group adsorption, the oxygen vacancies at the surface promote the fast and easy water and hydroxyl group adsorption, boosting the oxygen evolution reaction at the interface [56–58].

**Table 1:** Resistance calculation results from the equivalent circuit

Sample	R_s_ (Ω)	R_et_ (Ω)
Pristine (Air)	0.73	3677
Pristine (H_2_)	0.93	1580
SNT (Air)	1.46	1675
SNT (H_2_)	1.13	1263

Furthermore, Mott-Schottky analysis, as shown in Fig. 8e, was performed to examine the carrier concentration of each sample. The carrier concentration calculation using the results from the Mott-Schottky plot can be expressed by the following Equation (5):

5 $${N}_{D}=\frac{1.41\times {10}^{32}(\mathrm{cm}\times {F}^{-2}\times {V}^{-1}) }{{\varepsilon }_{r}\times {A}^{2}({\mathrm{cm}}^{4})\times \mathrm{slope}({F}^{-2}\times {V}^{-1})}$$

where *N*_D_ is the carrier concentration, *ε*_r_ is relative permittivity, *A* is the surface area of the electrode. The slope of the inverse square of the capacitance versus potential vs. RHE plot was examined. Detailed plots of each curve are displayed in Additional file 1: Fig. S7. And, the detailed parameters used in this calculation are stated in Additional file 1: Table S1. Each sample shows 1.64 × 10^19^ cm^−3^, 1.95 × 10^19^ cm^−3^, 5.11 × 10^19^ cm^−3^, and 1.39 × 10^20^ cm^−3^, respectively, which shows the increased carrier concentration due to the H_2_ annealing in both cases. Increased carrier concentration can be easily explained by Koeger-Vink notation below:

6 $${\text{O}}_{{\text{O}}} \to {\text{V}}_{{{\ddot{\text{o}}}}} + 2{\text{e}}^{ - }$$

where ${\text{V}}_{{{\ddot{\text{o}}}}}$ is vacant oxygen ion site, e^−^ is the electron [59]. Oxygen vacancies produced by H_2_ annealing generate the electrons increasing the carrier concentration of the matrix. The result of the reliability test is demonstrated in Fig. 8f. H_2_-treated S, N-doped TiO_2_ sample also exhibits outstanding reliability under the irradiation condition in the basic electrolyte. It maintains up to 95% of photocurrent density compared to the initial value for 100 h. Thus, these materials can be regarded as promising stable photoanodes materials.

To investigate the influence of H_2_ annealing on the surface adsorption capacity of water and hydroxyl groups, hydrophilicity difference under the air and H_2_ annealing condition was compared using the contact angle measurement. To prevent the undesirable water permeation in the photoelectrodes due to the nanostructure, the flat TiO_2_ thin films were prepared. The TiO_2_ thin films were annealed under an H_2_ atmosphere at 350 ℃, the same as the optimum annealing condition of S, N-doped TiO_2_ nanorod arrays. And, in order to compare the adsorption of the water molecule and hydroxyl groups, the contact angle of distilled water and 1M NaOH solution to both TiO_2_ thin films. The average contact angle between the H_2_-annealed TiO_2_ thin film and distilled water was approximately 38.57°, while the air-annealed TiO_2_ thin film showed 61.8° of contact angle. Reduced contact angle after the H_2_ annealing obviously shows that even 350 ℃ of H_2_ annealing makes TiO_2_ thin film more hydrophilic. A comparable result can be investigated in the case of 1 M NaOH solution which the electrolyte used for the PEC performance measurement. The average contact angle between the TiO_2_ thin film and 1 M NaOH decreases by 59.8% from approximately 30.23°–18.1° when applying the H_2_ annealing at TiO_2_ thin film. This is similar to the 62.4% reduction in the contact angle with water. To determine the interfacial free energy between the thin films and solutions, the interfacial energy was calculated using the following Eqs. (7) and (8):

7 $${\upgamma }_{s} = \left[ {{\upgamma }_{l} \left( {1 + \cos {\uptheta }} \right)^{2} } \right]/4$$

8 $${\upgamma }_{sl} = {\upgamma }_{s} - {\upgamma }_{l} \cos {\uptheta }$$

where γ_*s*_ is the surface free energy of the solid thin film, γ_*l*_ is the surface free energy of liquid, γ_*sl*_ is the surface free energy of between solid and liquid, and θ is the contact angle between solid and liquid [60]. The parameters for interfacial free energy calculation are stated in Table 2. H_2_ annealing at TiO_2_ thin film could stimulate the adsorption of H_2_O molecules and hydroxyl ions, which is verified by lowered interfacial free energy [56–58, 61]. Furthermore, when applying the H_2_ annealing, the interfacial free energy of 1 M NaOH solution changes further compared to the distilled water. It indicates that the adsorption effect of oxygen vacancy made by H_2_ annealing at the surface is much higher in the case of hydroxyl groups, being dissolved much amount in the NaOH solution.

**Table 2:** Interfacial free energy calculation

Sample	Angle (degree)	γ_L_ (mN/m)	γ_S_ (mN/m)	γ_SL_ (mN/m)
Air DI water	61.8	72.8	22.97	− 14.31
H_2_ DI water	38.57	72.8	25.83	− 21.30
Air NaOH	30.23	59.36	16.95	− 5.44
H_2_ NaOH	18.10	59.36	22.79	− 20.66

**Fig. 9:**
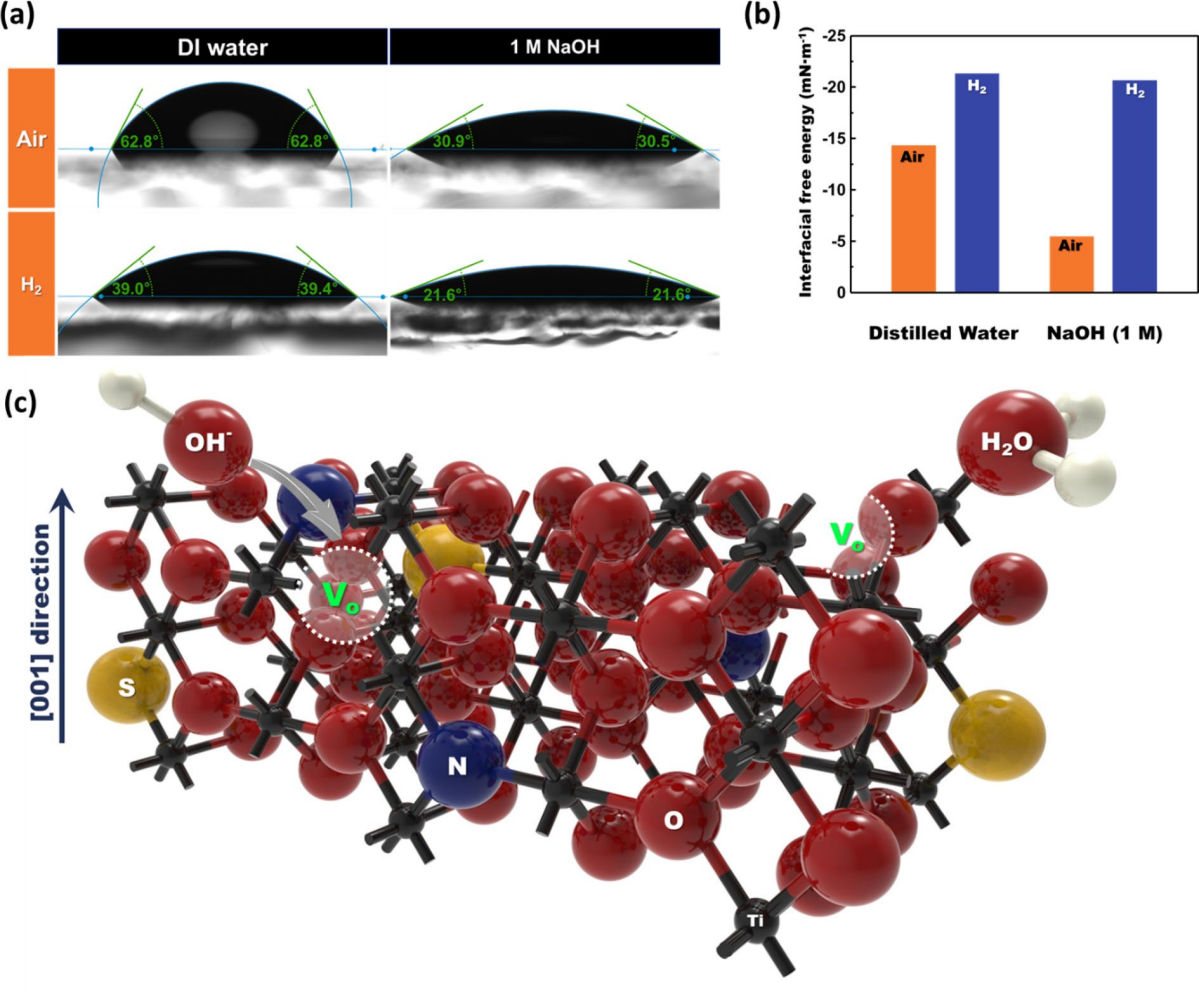
**a** The optical images captured by contact angle measurement system. The average contact angles were calculated using several images obtained by high speed camera. TiO_2_ thin films having 100 nm thickness were used. The contact angles between the films and DI water, 1 M NaOH solution were measured. **b** Comparison chart for Interfacial free energy. Interfacial free energies were calculated using contact angle and Young’s equation. **c** Schematics of surface reaction at the TiO_2_ surface. Oxygen vacancies at the surface, produced by H_2_ treatment, promote an easy adsorption of the water molecules and hydroxyl ions

Thus, the role of the optimized H_2_ annealing on S, N-doped TiO_2_ photoanode arranged into two aspects; increasing carrier concentration, promoting the adsorption of water molecules and hydroxyl ions. The carrier concentration increases as shown in Eq. (6) as the S, N-doped TiO_2_ is reduced due to H_2_ annealing and oxygen atoms on the TiO_2_ surface escape. However, H_2_ annealing at a high temperature of 550 °C or higher increases the carrier concentration too much, causing S, N-doped TiO_2_ to lose its semiconducting properties and become metallically conductive. The expected behavior of the water molecules and hydroxyl ions on the surface of S, N-doped TiO_2_ is illustrated in Fig. 9c. Oxygen vacancies on the surface created by the H_2_ annealing lower the interfacial free energy between the TiO_2_ and the electrolyte, making TiO_2_ more hydrophilic. The hydrophilic surface has more active adsorption/desorption of H_2_O and hydroxyl groups. In other words, more reactants can participate in each oxygen evolution reaction step making the overall OER reaction easier to proceed. Furthermore, the surface disorder leads to the dopant loss at the photoanodes’ surface [62]. Therefore, the surface disorder ruins the total PEC performance of TiO_2_ photoanodes, especially for the doped TiO_2_ photoanodes. This work emphasizes the importance of the optimum condition of H_2_ annealing for photoelectrode. In the case of photoelectrodes, the charge separation and transport are important rather than the photocatalyst, as each carrier migrates toward the counter electrode and semiconductor/electrolyte interface, respectively. Therefore, the charge trap of defect level influences further to the photoelectrodes rather than the bandgap narrowing. Too many defect levels due to the surface disorder promote the charge trap and recombination at the surface, which hinders the electron separation and migration.

## Conclusions

Our results clearly show the effects of optimum H_2_ annealing condition on the S, N-doped TiO_2_ based photoanodes. The notable advance in PEC performance for TiO_2_-based photoanodes was achieved by the optimum H_2_ annealing condition. The effective PEC performance can be explained by the 3.04 mA·cm^−2^ of photocurrent density, 4.25 times higher than the pristine TiO_2_. 78.16% of IPCE at 380 nm wavelength confirms the prominence of this material at the near-UV spectral range. Moreover, the outstanding PEC performance was maintained up to 95% and for 100 h, which shows great reliability of the photoanodes in this work. The optimum H_2_ annealing condition for the S, N-doped TiO_2_ photoanodes was established by examining the crystallographic, bandgap, and elemental analysis under the various H_2_ annealing temperature. In a good agreement with the previous works related to the anatase phase TiO_2_ based black TiO_2_ photocatalysts, the black color due to the surface disorder induces at the rutile phase TiO_2_ photoanodes. The narrowed bandgap due to the defect level also occurs. But, too much amount of surface disorder and dopant loss at the photoanodes’ surface interrupts the PEC performance. Under the optimum H_2_ annealing condition, the increment of carrier concentration was checked by Mott-Schottky analysis. Increased hydrophilicity under the optimum condition was displayed by the contact angle analysis and calculated interfacial free energy. Furthermore, by comparing the interfacial free energy, the H_2_ annealing influences further in the case of 1 M NaOH electrolyte, which has much higher hydroxyl ion concentration than the distilled water. Thus, H_2_ annealing much effectively affects the hydroxyl ion adsorption rather than the water molecule. Without the support of the other materials, highly effective TiO_2_ photoanodes were achieved via a simple hydrothermal process and annealing under the optimum H_2_ atmosphere. Therefore, facile mass production is expected by this simple all solution-based process. Furthermore, these TiO_2_ only photoanodes promise further improvement by applying the other effective strategies such as heterojunction and co-catalyst.

## Supplementary Information

**Additional file 1: Figure S1. **The photocurrent density of S, N-doped TiO_2_ photoanodes under various annealing conditions. The photocurrent density of 350 ℃ air-annealed S, N-doped TiO_2_ photoanode was set as the reference, displayed as the gray dotted line. **Figure S2. **XPS spectra of Ti 2*p* for H_2_ heat treatment condition. **Figure S3. **Nanostructure of the reference samples investigated by SEM. Pristine TiO_2_ nanorod arrays annealed under (a) ambient air and (b) H_2_ at 350 ℃. (c) S, N-doped TiO_2_ nanorod arrays annealed under ambient air at 350 ℃. **Figure S4. **Ti 2*p* XPS spectra of the optimum and reference samples. **Figure S5. **(a, b) N 1*s* and (c, d) S 2*p* XPS spectra of the optimum and reference samples. **Figure S6. **The results of linear sweep voltammetry(LSV) including non-annealed samples. LSV curves for (a) pristine TiO_2_ photoanodes and (b) S, N-doped TiO_2_ photoanodes. **Figure S7. **Graphs showing the Mott-Schottky curve fitting for carrier concentration calculation. Mott-Schottky curve fitting for pristine TiO_2_ nanorod arrays annealed under (a) ambient air and (b) H_2_ atmosphere. Fitting was also operated to the Mott-Schottky curve of S, N-doped TiO_2_ nanorod arrays annealed under (c) ambient air and (d) H_2_ atmosphere. **Table S1.** Carrier concentration calculation.

## Data Availability

Not applicable.

## Abbreviations

PEC: Photoelectrochemical
FTO: Fluorine-doped tin oxide
GIXRD: Glazing incident X-ray diffractometer
FE-SEM: Field emission scattering electron microscopy
TEM: Transmission electron microscopy
EDS: Energy dispersive spectroscopy
XPS: X-ray photoelectron spectrometer
LSV: Linear sweep voltammetry
EIS: Electrochemical impedance spectroscopy
IPCE: Incident photon-to-current efficiency
M-S: Mott-Schottky
XRD: X-ray diffraction
SEM: Scanning electron microscope
