# Supplementary material for: Regulating the surface of anion-doped TiO2 nanorods by hydrogen annealing for superior photoelectrochemical water oxidation
Source: Nano Converg. 2022 Jul 19;9:33. doi: 10.1186/s40580-022-00323-9 (PMC9296745; doi:10.1186/s40580-022-00323-9)
Supplement: Supplementary file 1 — Additional file 1: Figure S1. The photocurrent density of S, N-doped TiO2 photoanodes under various annealing conditions. The photocurrent density of 350 ℃ air-annealed S, N-doped TiO2 photoanode was set as the reference, displayed as the gray dotted line. Figure S2. XPS spectra of Ti 2p for H2 heat treatment condition. Figure S3. Nanostructure of the reference samples investigated by SEM. Pristine TiO2 nanorod arrays annealed under (a) ambient air and (b) H2 at 350 ℃. (c) S, N-doped TiO2 nanorod arrays annealed under ambient air at 350 ℃. Figure S4. Ti 2p XPS spectra of the optimum and reference samples. Figure S5. (a, b) N 1s and (c, d) S 2p XPS spectra of the optimum and reference samples. Figure S6. The results of linear sweep voltammetry(LSV) including non-annealed samples. LSV curves for (a) pristine TiO2 photoanodes and (b) S, N-doped TiO2 photoanodes. Figure S7. Graphs showing the Mott-Schottky curve fitting for carrier concentration calculation. Mott-Schottky curve fitting for pristine TiO2 nanorod arrays annealed under (a) ambient air and (b) H2 atmosphere. Fitting was also operated to the Mott-Schottky curve of S, N-doped TiO2 nanorod arrays annealed under (c) ambient air and (d) H2 atmosphere. Table S1. Carrier concentration calculation. [file 40580_2022_323_MOESM1_ESM.docx]

**Regulating the Surface of Anion-Doped TiO_2_ Nanorods by Hydrogen Annealing for Superior Photoelectrochemical Water Oxidation**

Jongseong Park ^‡,1^, Seonyong Lee ^‡,1^, Tae Hyung Lee ^1^, Changyeon Kim ^1^, Sang Eon Jun ^1^, Ji Hyun Baek ^1^, Jae Young Kim ^1^,

1. Department of Materials Science and Engineering, Research Institute of Advanced Materials, Seoul National University, Gwanak-ro 1, Seoul, 08826, Republic of Korea
2. Department of Electrical and Computer Engineering, University of Toronto, 35 St. George Street, Toronto, Ontario, M5S 1A4, Canada
3. School of Chemical Engineering and Materials Science, Chung-Ang University, 84 Heukseok-ro, Dongjak-gu, Seoul 06974, Korea
4. Advanced Institute of Convergence Technology, Seoul National University, Suwon, 16229, Republic of Korea

- Corresponding author: Sang Hyun Ahn, Ho Won Jang

Tel.: +82-2-880-1720

E-mail address: shahn@cau.ac.kr, hwjang@snu.ac.kr

**Figure captions:**

**Fig. S1.** The photocurrent density of S, N-doped TiO_2_ photoanodes under various annealing conditions. The photocurrent density of 350 ℃ air-annealed S, N-doped TiO_2_ photoanode was set as the reference, displayed as the gray dotted line.

**Fig. S2.** XPS spectra of Ti 2*p* for H_2_ heat treatment condition.

**Fig. S3.** Nanostructure of the reference samples investigated by SEM. Pristine TiO_2_ nanorod arrays annealed under (a) ambient air and (b) H_2_ at 350 ℃. (c) S, N-doped TiO_2_ nanorod arrays annealed under ambient air at 350 ℃.

**Fig. S4.** Ti 2*p* XPS spectra of the optimum and reference samples.

**Fig. S5.** (a, b) N 1*s* and (c, d) S 2*p* XPS spectra of the optimum and reference samples.

**Fig. S6.** The results of linear sweep voltammetry(LSV) including non-annealed samples. LSV curves for (a) pristine TiO_2_ photoanodes and (b) S, N-doped TiO_2_ photoanodes.

**Fig. S7.** Graphs showing the Mott-Schottky curve fitting for carrier concentration calculation. Mott-Schottky curve fitting for pristine TiO_2_ nanorod arrays annealed under (a) ambient air and (b) H_2_ atmosphere. Fitting was also operated to the Mott-Schottky curve of S, N-doped TiO_2_ nanorod arrays annealed under (c) ambient air and (d) H_2_ atmosphere.

**Tables:**

**Table S1.** Carrier concentration calculation

**Fig. S1.**


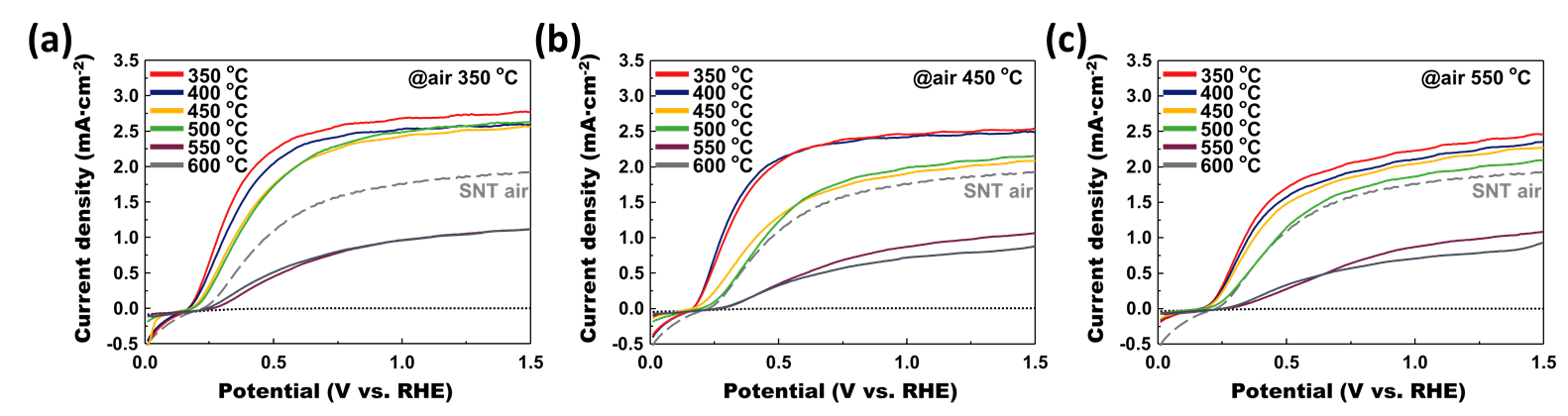


**Fig. S2.**


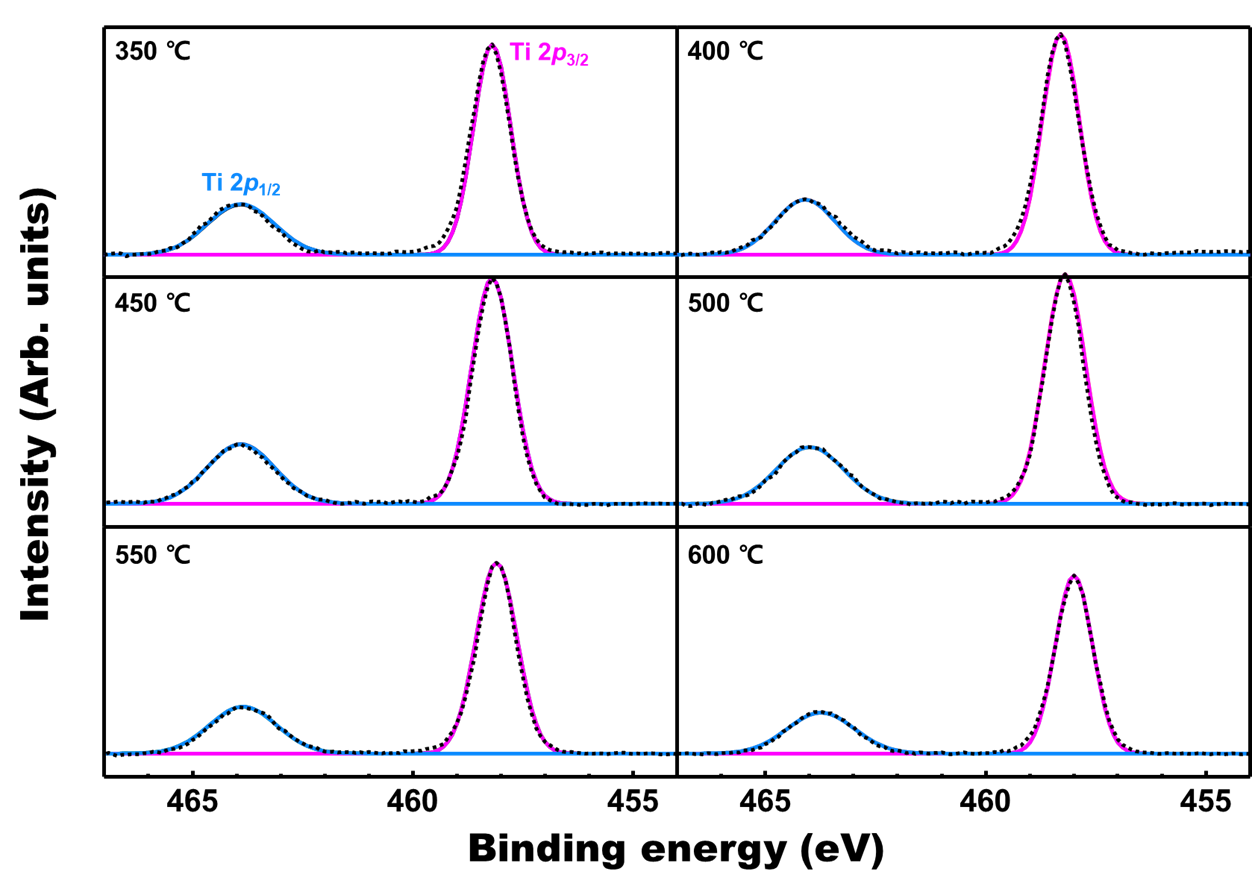


**Fig. S3.**


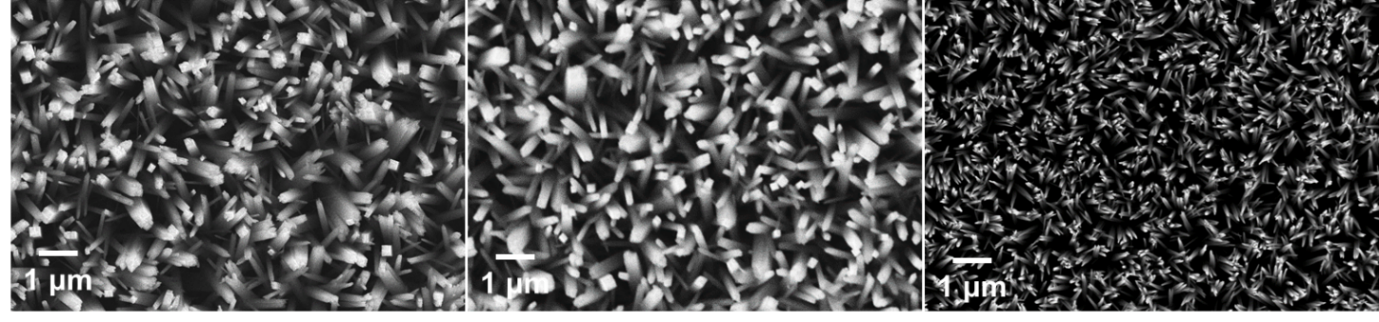


**Fig. S4.**


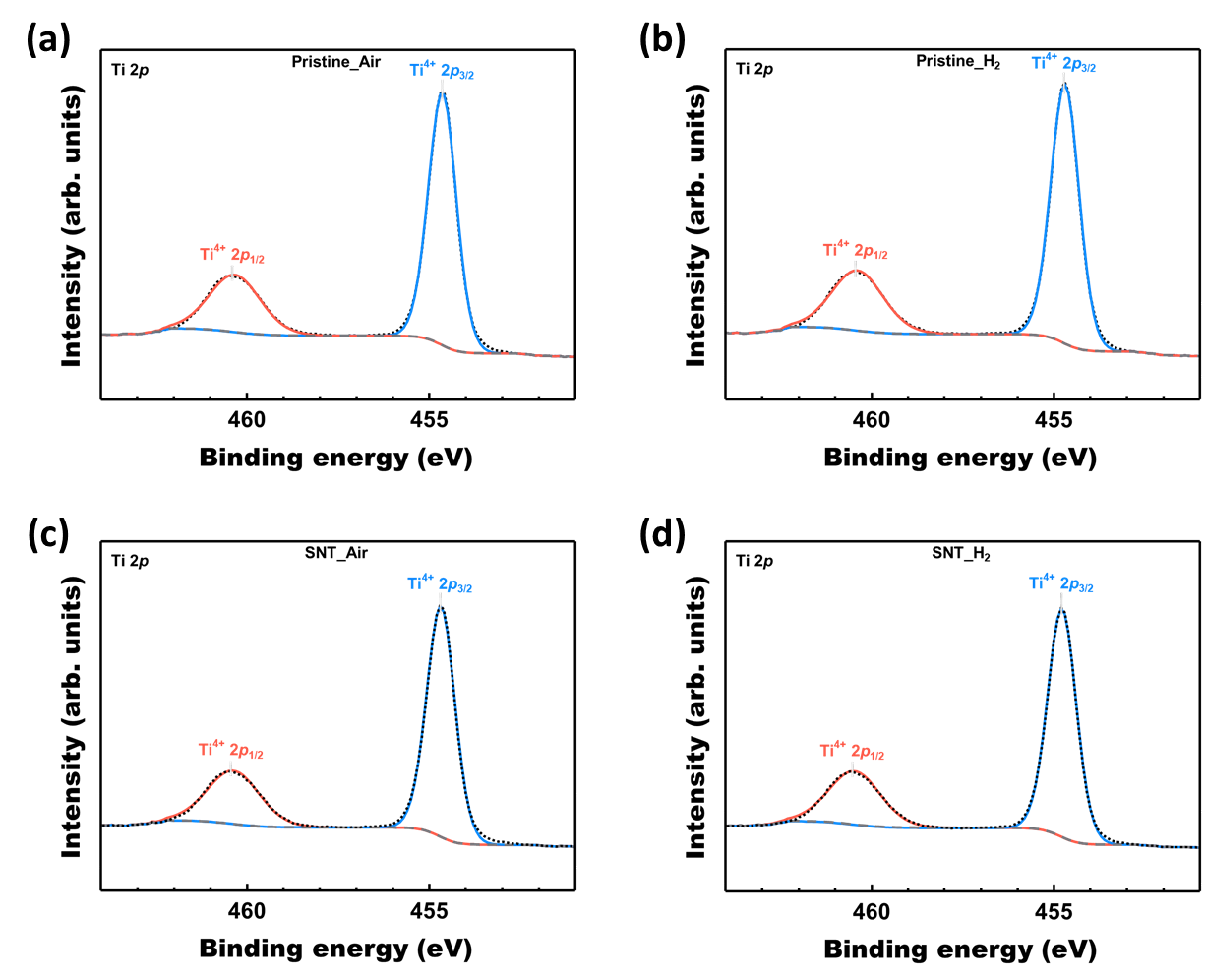


**Fig. S5.**

**
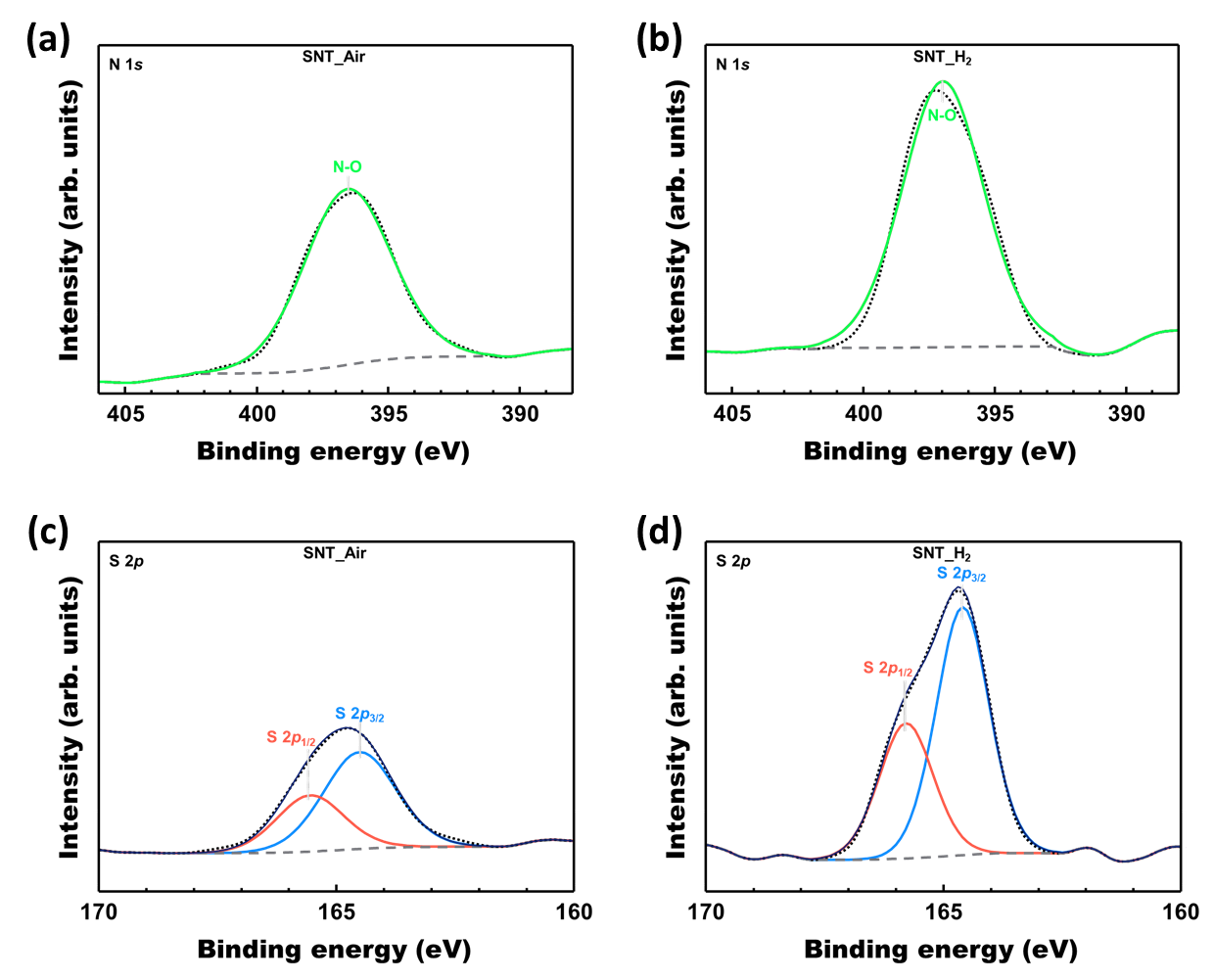
**

**Fig. S6.**


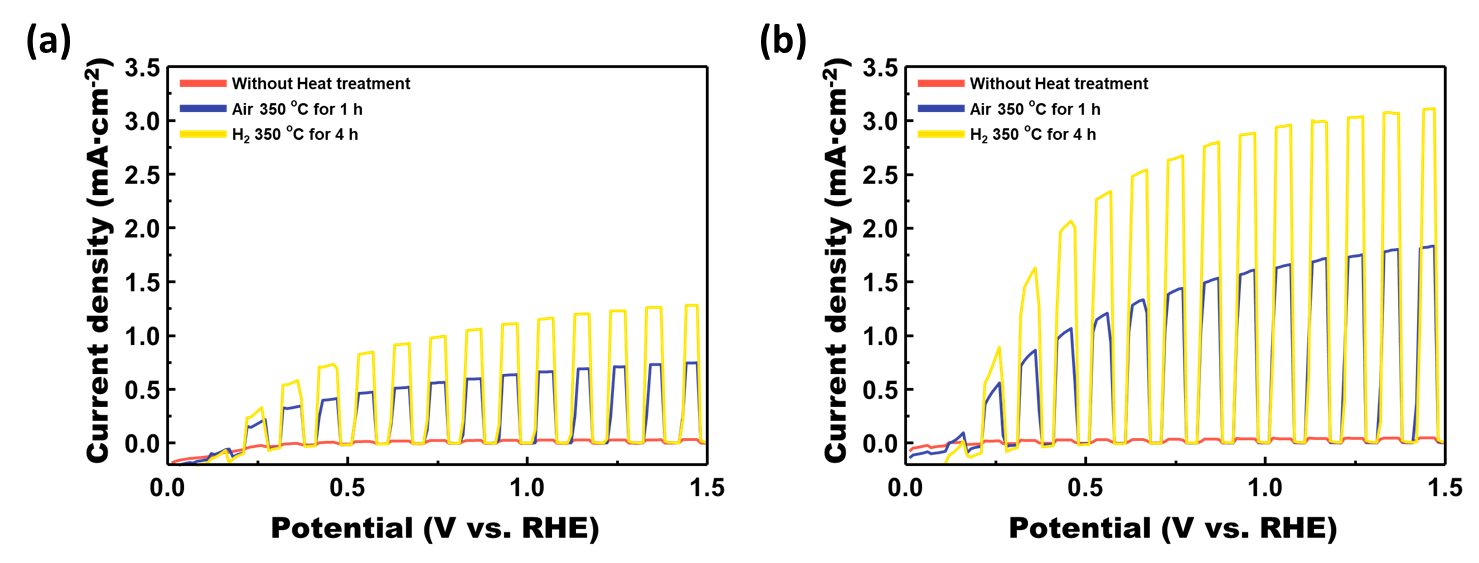


**Fig. S7.**


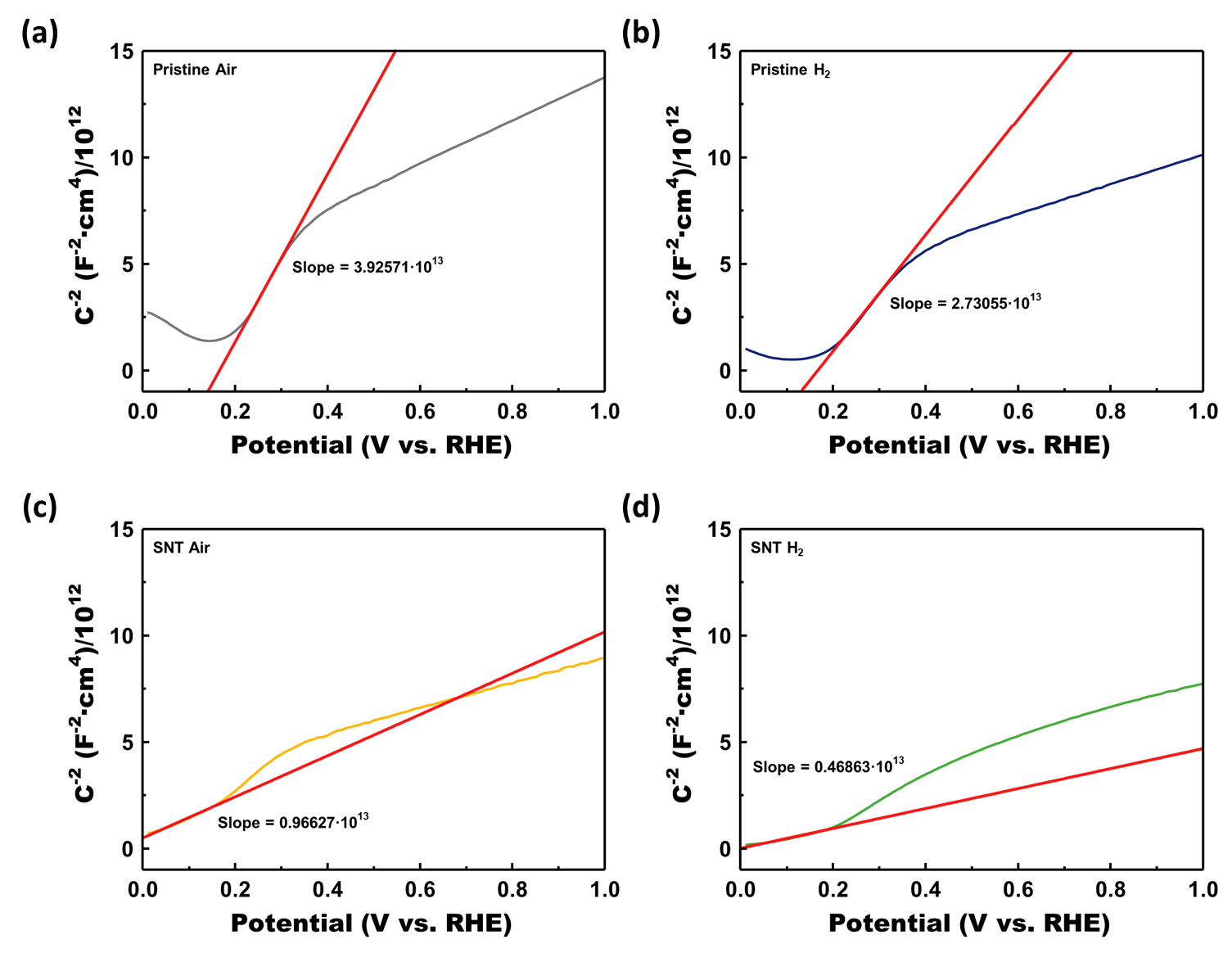


**Table S1.** Carrier concentration calculation

| Sample | $\frac{\boldsymbol{d}\boldsymbol{C}^{\boldsymbol{-2}}}{\boldsymbol{dE}}$ (*F* ^-2^·*V* ^-1^) | $\boldsymbol{\varepsilon}_{\boldsymbol{r}}$ | A (cm^2^) | $\boldsymbol{N}_{\boldsymbol{D}}$ (cm^-3^) |
| --- | --- | --- | --- | --- |
| Pristine  Air | 3.93 $\times$ 10^13^ | 170 | 0.03587 | 1.64 $\times$ 10^19^ |
| Pristine  H_2_ | 2.73 $\times$ 10^13^ | 170 | 0.03950 | 1.95 $\times$ 10^19^ |
| SNT  Air | 9.66 $\times$ 10^12^ | 170 | 0.04099 | 5.11 $\times$ 10^19^ |
| SNT  H_2_ | 4.69 $\times$ 10^12^ | 170 | 0.03569 | 1.39 $\times$ 10^20^ |
